# NLK is required for Ras/ERK/SRF/ELK signaling to tune skeletal muscle development by phosphorylating SRF and antagonizing the SRF/MKL pathway

**DOI:** 10.1038/s41420-021-00774-9

**Published:** 2022-01-10

**Authors:** Shang-Ze Li, Ze-Yan Zhang, Jie Chen, Ming-You Dong, Xue-Hua Du, Jie Gao, Qi-Peng Shu, Chao Li, Xin-Yi Liang, Zhi-Hao Ding, Run-Lei Du, Junli Wang, Xiao-Dong Zhang

**Affiliations:** 1 Hubei Key Laboratory of Cell Homeostasis, College of Life Sciences, Wuhan University, 430072 Wuhan, Hubei China; 2 School of Medicine, Chongqing University, 400030 Chongqing, China; 3 Reproductive genetics laboratory, Affiliated hospital of Youjiang Medical University for Nationalities, 533000 Baise, Guangxi China

**Keywords:** Cell signalling, Growth factor signalling, Cell division

## Abstract

Serum response factor (SRF) regulates differentiation and proliferation by binding to RhoA-actin-activated MKL or Ras-MAPK-activated ELK transcriptional coactivators, but the molecular mechanisms responsible for SRF regulation remain unclear. Here, we show that Nemo-like kinase (NLK) is required for the promotion of SRF/ELK signaling in human and mouse cells. NLK was found to interact with and phosphorylate SRF at serine residues 101/103, which in turn enhanced the association between SRF and ELK. The enhanced affinity of SRF/ELK antagonized the SRF/MKL pathway and inhibited mouse myoblast differentiation in vitro. In a skeletal muscle-specific *Nlk* conditional knockout mouse model, forming muscle myofibers underwent hypertrophic growth, resulting in an increased muscle and body mass phenotype. We propose that both phosphorylation of SRF by NLK and phosphorylation of ELKs by MAPK are required for RAS/ELK signaling, confirming the importance of this ancient pathway and identifying an important role for NLK in modulating muscle development in vivo.

## Introduction

SRF is a member of the myocyte enhancer factor-2 (MEF2) family of MADS-box transcription factors, including MCM1, Agamous, and Deficiens, that precisely orchestrates the growth and development of skeletal muscle [1]. Stimulation with serum or a growth factor changes the status of cell growth and the expression of large numbers of genes, which is dependent on SRF and a cis-acting element in the promoter termed the serum response element (SRE). SRF modulates cell proliferation and morphology by binding different coactivator ETS-like proteins (ELKs) and myocardin-like factors (MKLs) to induce the expression of specific types of genes [2]. The immediate early genes (IEGs) induced by serum or growth factors, such as *EGR1, C-FOS,* and *TCF7L2*, are largely dependent on SRF through binding to members of the SRF coactivator ternary complex factor (TCF) family, including ELK1, ELK4 (SAP1) and NET (ELK3, SAP2, or ERP), which are phosphorylated and activated by mitogen-activated protein kinase (MAPK) in the RAS-ERK axis [3–5]. SRF regulates muscle differentiation genes by binding members of another coactivator family, MKL1 and MKL2 (also called myocardin-related transcription factors (MRTFs)), in response to the Rho-actin pathway [6]. The interactions of ELK1 and MKLs with SRF have been found to be mutually exclusive due to competitive binding to the DNA-binding domain of SRF [7, 8]. Therefore, the balance between these two pathways is precisely controlled and affects cell proliferation and adhesive properties [9, 10].

The binding between ELK1 and SRF is controlled by phosphorylation of ELK1 through the RAS-ERK axis. The phosphorylation of ELK1 induced by platelet-derived growth factor (PDGF) results in an enhanced association between ELK1 and SRF [10, 11]. G-actin binds to MKL1 and maintains the cytosolic localization of MKL1. Activation of Rho induces G-actin polymerization into F-actin and MKL1 release into the nucleus for binding to SRF as well as subsequent activation of target genes [12]. MKL1 phosphorylation is not a prerequisite for the control of its nuclear accumulation [13].

Previous studies have shown that in smooth muscle cells, PDGF induces SRF-associated cofactor exchange [10]. Using integrated SRF chromatin immunoprecipitation sequencing (ChIP-seq) and Hi-C data, Francesco et al. also found that competition between TCFs and MKLs for SRF determined the balance between antagonistic proliferative and contractile programs [9]. Although they identified cofactor competition as a general feature of SRF regulation, how the strict competition mechanism underlies balance regulation remains unclear.

Post-translational modification (PTM) plays an important role in protein activity regulation. Previous studies have shown that SRF phosphorylation at amino acids 77–85 and 103 greatly reduces the DNA-binding ability of SRF. However, none of these mutations affects the ability of SRF to interact with ELK1. There are no phosphorylation sites reported to regulate the balance between SRF/ELK and SRF/MKL [14].

Here, we characterized the function of NLK in the ELK1/SRF pathway. We reported the dramatic positive regulation of ELK1/SRF signaling by NLK. NLK ablation abolished ELK1/SRF signaling, which means that NLK is required for ELK1/SRF activation. Moreover, SRF, as a newly identified substrate of NLK, was characterized. Serine residue 101/103 phosphorylation of SRF increased the binding of SRF to ELK1 and simultaneously decreased the affinity of SRF for MKL1. Skeletal muscle-specific NLK deletion in mice produced increased skeletal muscle development and delayed differentiation. Our data suggest that NLK is vital in modulating the balance between SRF/MKL1 and SRF/ELK1 through SRF phosphorylation to control skeletal muscle development and differentiation.

## Results

### NLK is a potent positive regulator of Ras-ERK transcriptional activation

**Fig. 1:**
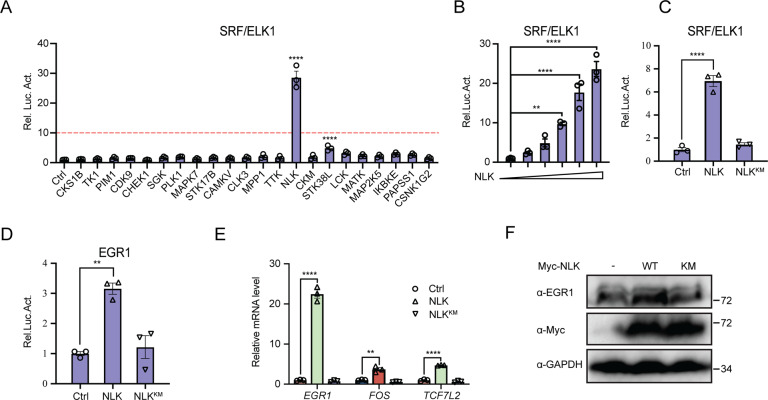
NLK is a promoter of the SRF/ELK signaling pathway. **A** SRF/ELK pathway screening with a luciferase assay using a kinase library. SRF/ELK reporter (100 ng) was cotransfected with the indicated kinase plasmids (400 ng) into HEK293T cells for 36 h, followed by analysis with a luciferase kit assay (*n* = 2). **B** NLK promotes SRF/ELK signaling in a dose-dependent manner. SRF/ELK reporter (100 ng) was cotransfected with increasing concentrations of a Flag-NLK expression plasmid (0, 25, 50, 100, 200, or 400 ng) into HEK293T cells for 36 h, followed by analysis with a luciferase kit assay (*n* = 3). **C**, **D** Effects of NLK or NLK^KM^ on SRF/ELK (**C**) or EGR1 (**D**) luciferase reporter activity in HEK293T cells. The SRF/ELK reporter (100 ng) or EGR1 reporter (100 ng) was cotransfected with an NLK or NLK^KM^ plasmid (200 ng) into HEK293T cells for 36 h, followed by analysis with a luciferase kit assay (*n* = 3). **E** Real-time PCR showing the effects of NLK or NLK^KM^ plasmid (200 ng) on *EGR1, FOS,* and *TCF7L2* gene transcription in HEK293T cells. The real-time PCR values were normalized to the *GAPDH* values (*n* = 3). **F** Immunoblotting showing the effects of NLK or NLK^KM^ on the protein level of EGR1 using the indicated antibodies in HEK293T cells. GAPDH was used as a loading control. Data are representative of three independent experiments and presented as the mean ± SEM. Statistical significance was analyzed by ANOVA (*p < 0.05, **p < 0.01, ***p < 0.001, ****p < 0.0001). Ctrl control, NLK^KM^ Mutation of lysine 167 to methionine. Source data (**A–F**) are provided as a source data file.

Ras-ERK signaling, which is conducted by the ELK/SRF transcriptional complex, is critical in the control of proliferation, invasion, and metastasis. To characterize the kinase potentially regulating Ras-ERK signaling, we screened ~200 kinases using an ELK/SRF luciferase reporter. NLK dramatically increased ELK/SRF luciferase activity in HEK293T cells (Fig. 1A). Although NLK was not the only kinase that could induce ELK/SRF luciferase activation, further confirmation indicated that NLK potently promoted ELK/SRF luciferase reporter activation in a dose-dependent manner (Fig. 1B). To determine the roles of kinase activity in ELK/SRF signaling, NLK^KM^, a kinase-dead NLK mutant with replacement of lysine 167 with methionine, was employed [15, 16]. Both an ELK/SRF reporter and an EGR1 (an ELK/SRF target gene) reporter were significantly activated by NLK but not NLK^KM^, which means that NLK kinase activity is required for ELK/SRF signaling (Fig. 1C, D). To further verify the function of NLK, we next performed real-time PCR to test ELK/SRF downstream genes. The results demonstrated that NLK but not NLK^KM^ promoted the transcription of classic ELK/SRF target genes, including *EGR1*, *FOS,* and *TCF7L2* (Fig. 1E). An upregulated EGR1 protein level was also observed in the presence of NLK by immunoblot analysis (Fig. 1F). These data suggested that NLK markedly enhanced ELK1-SRF signaling.

### Deficiency in NLK reduces ELK/SRF signaling in human and mouse cells

**Fig. 2:**
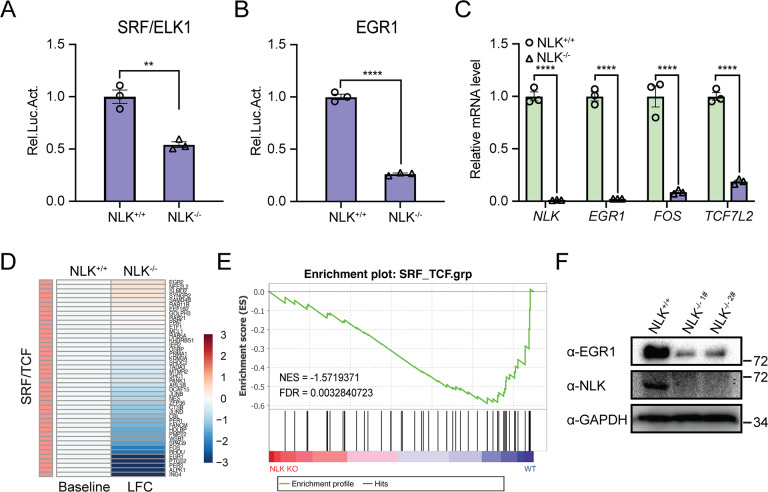
Loss of NLK blocks SRF/ELK signaling. **A**, **B** Luciferase assays for SRF/ELK reporter (**A**) or EGR1 reporter (**B**) in wild-type and NLK-deficient HCT116 cells. SRF/ELK reporter (200 ng) or EGR1 reporter (200 ng) was transfected into NLK-deficient HCT116 cells for 36 h, followed by analysis with a luciferase kit assay (*n* = 3). **C** Real-time PCR showing the gene transcription levels of *NLK, EGR1, FOS,* and *TCF7L2* in wild-type and NLK-deficient HCT116 cells. The real-time PCR values were normalized to the *GAPDH* values (*n* = 3). **D** Heatmap showing the differentially expressed genes related to SRF/TCF signaling between wild-type and NLK-deficient HCT116 cells. **E** GSEA showing SRF/TCF signaling enrichment between wild-type cells and NLK-deficient HCT116 cells. **F** Immunoblotting showing the protein levels of EGR1 and NLK in NLK-deficient HCT116 cells using the indicated antibodies. GAPDH was used as a loading control. Data are representative of three independent experiments and presented as the mean ± SEM. Statistical significance was analyzed by Student’s *t*-test or ANOVA (**p < 0.01, ****p < 0.0001). LFC log fold change, NES normalized enrichment score, FDR false discovery rate. Source data (**A–F**) are provided as a source data file.

To examine the regulation of SRF/ELK by NLK in a more physiological context, we used previously generated HCT116/NLK^−/−^ cells [17]. We first assessed the effects of NLK deficiency on the ELK/SRF pathway using luciferase reporter assays. The results showed that NLK-deficient cells exhibited markedly decreased ELK/SRF and EGR1 luciferase reporter activity compared with parental HCT116 cells (Fig. 2A, B). We further assessed the effects of NLK deficiency on ELK/SRF activation by evaluating the mRNA levels of *EGR1*, *FOS,* and *TCF7L2* and found that NLK deficiency almost completely blocked the transcription of these genes, especially *EGR1* (Fig. 2C). Gene set enrichment analysis (GSEA) using previous RNA-sequencing (RNA-seq) results [17] revealed significantly negative enrichment of SRF/TCF target genes upon NLK knockout, and the mRNA levels of most SRF/TCF target genes were downregulated in NLK-deficient HCT116 cells, as shown in the heatmap (Fig. 2D, E). Moreover, an immunoblotting experiment showed that the protein level of ERG1 was downregulated in NLK-deficient cells (Fig. 2F).

**Fig. 3:**
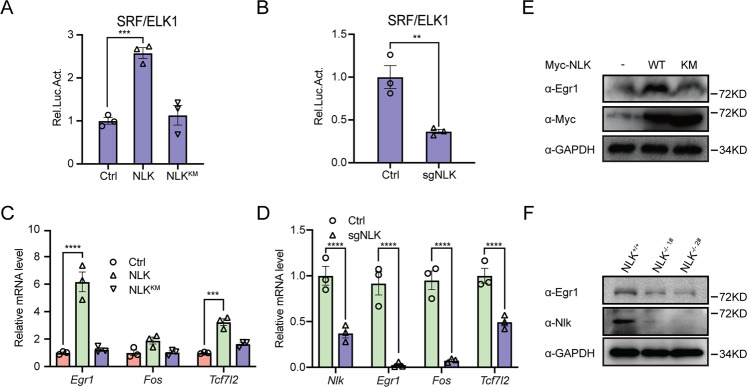
NLK regulates SRF/ELK signaling in C2C12 cells. **A** Luciferase assays showing the effects of NLK or NLK^KM^ on SRF/ELK in C2C12 cells. SRF/ELK reporter (200 ng) was cotransfected with an NLK or NLK^KM^ plasmid (200 ng) into C2C12 cells for 36 h, followed by analysis with a luciferase kit assay (*n* = 3). **B** Luciferase assays showing the effects of NLK deficiency on SRF/ELK in C2C12 cells. **C** Real-time PCR showing the effects of NLK or NLK^KM^ plasmid (200 ng) on *Egr1, Fos,* and *Tcf7l2* gene transcription in C2C12 cells. The real-time PCR values were normalized to the *Gapdh* values (*n* = 3). **D** Real-time PCR showing the gene transcription levels of *Nlk, Egr1, Fos,* and *Tcf7l2* in wild-type and NLK-deficient C2C12 cells. The real-time PCR values were normalized to the *Gapdh* values (*n* = 3). **E** Immunoblotting showing the effects of NLK or NLK^KM^ on Egr1 protein levels using an anti-Egr1 antibody in HEK293T cells. GAPDH was used as a loading control. **F** Immunoblotting showing the protein levels of Egr1 and Nlk in wild-type and Nlk-deficient C2C12 cells using anti-Nlk and anti-Egr1 antibodies. GAPDH was used as a loading control. Data are representative of three independent experiments and presented as the mean ± SEM. Statistical significance was analyzed by ANOVA (**p < 0.01, ***p < 0.001, ****p < 0.0001). Ctrl control. Source data (**A–F**) are provided as a source data file.

Next, we determined whether the regulation of ELK/SRF by NLK has extensive adaptability in C2C12 cells, which are derived from mice and are myoblasts [18]. C2C12 cells with CRISPR-mediated *Nlk* deletion and *NLK-* and *NLK*^*KM*^-overexpressing C2C12 cells were first obtained. Luciferase reporter assays for ELK/SRF activation in these cell lines were performed. The results showed that NLK, not NLK^KM^ activated the ELK/SRF pathway (Fig. 3A); however, NLK depletion inhibited ELK/SRF reporter activation (Fig. 3B). Consistently, NLK overexpression increased the transcription of the ELK/SRF target genes *Egr1*, *Fos,* and *Tcf7l2* and Egr1 protein level (Fig. 3C, E). In contrast, *Nlk*-ablation-C2C12 cells significantly blocked *Egr1*, *Fos* and *Tcf7l2* gene transcription and Egr1 protein expression (Fig. 3D, F). Taken together, these suggest NLK is required for Ras-ERK-ELK-SRF signaling in human and mouse cells.

### NLK interacts with and phosphorylates SRF

**Fig. 4:**
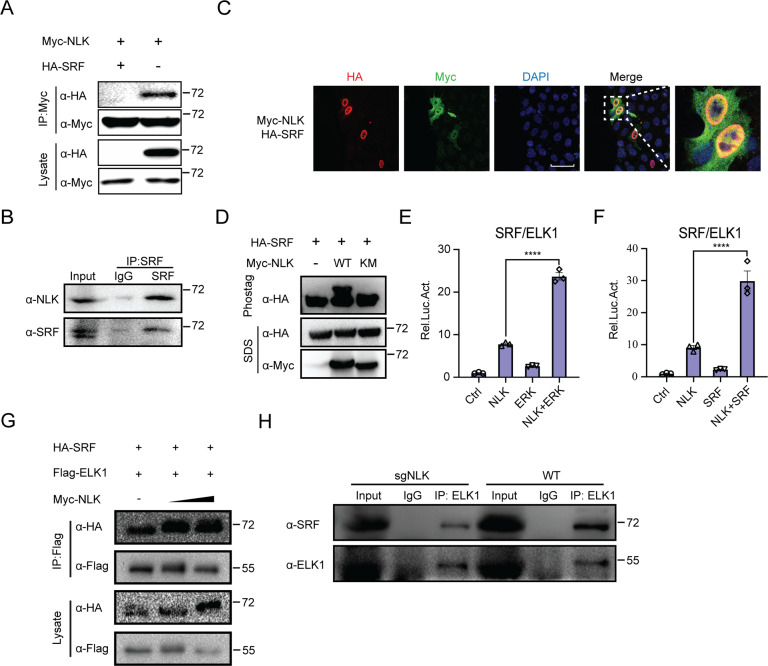
NLK enhanced the interaction between SRF and ELK by phosphorylating SRF. **A** Exogenous coimmunoprecipitation analysis showing the interaction between NLK and SRF. HEK293T cells were cotransfected with HA-SRF and Myc-NLK. Coimmunoprecipitation and immunoblotting were performed with the indicated antibodies. **B** Endogenous coimmunoprecipitation of C2C12 cell lysates performed with an anti-SRF antibody. Immunoblot analysis was performed with anti-NLK and anti-SRF antibodies. **C** Immunofluorescence analysis of the colocalization of exogenous NLK or NLK^KM^ and SRF. Myc-NLK (green) and HA-SRF (red) were stained using anti-Myc and anti-HA antibodies. The nuclei were stained with DAPI (blue). Images were obtained by fluorescence microscopy. Scale bar, 50 μm. **D** Phos-tag^TM^ SDS-PAGE showing the phosphorylation status of SRF in the presence of NLK or NLK^KM^ in HEK293T cells. HA-SRF was cotransfected with Myc-NLK- or Myc-NLK^KM^-overexpression plasmids into HEK293T cells, which were lysed 36 h after transfection. Proteins were separated by SDS-PAGE containing Phos-tag^TM^ and general SDS-PAGE with anti-HA and anti-Myc antibodies. The upper shifted band represents phosphorylated SRF protein. The bottom band represents the unphosphorylated form of SRF. **E**, **F** Luciferase assays showing the synergistic effects of NLK and ERK (**E**) or SRF (**F**) on SRF/ELK signaling in HEK293T cells. SRF/ELK reporter (100 ng) was cotransfected with the NLK and ERK or SRF plasmids (200 ng) into HEK293T cells for 36 h, followed by analysis with a luciferase kit assay (*n* = 3). **G** Coimmunoprecipitation analysis showing the effects of NLK on the interaction between ELK1 and SRF. HEK293T cells were cotransfected with HA-SRF, Flag-ELK1, and Myc-NLK. Coimmunoprecipitation and immunoblotting were performed with the indicated antibodies. **H** Coimmunoprecipitation of endogenous proteins in wild-type and NLK-knockdown C2C12 cells performed with an anti-ELK1 antibody in the presence of TPA. Immunoblotting was performed with anti-ELK1 and anti-SRF antibodies. Data are representative of three independent experiments and presented as the mean ± SEM. Statistical significance was analyzed by ANOVA (****p < 0.0001). Ctrl control, WT wild-type, KM NLK^KM^ mutant. Source data (**A–H**) are provided as a source data file.

ERKs phosphorylate ELKs and thus give rise to increased binding between ELKs and SRF and subsequent transcription of ELK/SRF target genes. NLK is a noncanonical MAPK family member that is highly similar to Erk2 [15, 19]. Accordingly, we predicted that NLK can redundantly bind and phosphorylate ELK1. However, we did not detect the interaction. Interestingly, coimmunoprecipitation analysis demonstrated a strong interaction between NLK and SRF (Fig. 4A). Endogenous coimmunoprecipitation experiments further confirmed the physiological interaction between NLK and SRF in C2C12 cells (Fig. 4B). To further verify the association between NLK and SRF, we performed an immunofluorescence experiment to analyze the colocalization. We observed that overexpressed NLK colocalized very well with SRF in the nucleus (Fig. 4C). Thus, we hypothesized that the NLK-SRF axis is parallel to rather than redundant with the ERK/ELK axis, which results in phosphorylated SRF and ELK, respectively, and that both are required for subsequent SRF/ELK binding and transcriptional activation of downstream genes. To this end, we first carried out a Phos-tag^TM^ SDS-PAGE assay to determine whether NLK gives rise to SRF phosphorylation. As shown in Fig. 4D, NLK not NLK^KM^ resulted in a shift observed by Phos-tag^TM^ SDS-PAGE, which indicated that NLK gives rise to SRF phosphorylation in a manner dependent on NLK kinase activity. To determine whether NLK and ERK function upstream of SRF and ELKs, we tested the synergistic effect of SRF/NLK or ERK/NLK on SRF/ELK transcriptional activation. The results showed that NLK synergized with both SRF and ERK to activate SRF/ELK transcription (Fig. 4E, F). Increased interaction between SRF and ELK, which was dramatically strengthened by coexpression of NLK, was observed (Fig. 4G). In contrast, NLK deficiency decreased the binding between SRF and ELK after 12-O-tetradecanoyl phorbol-13-acetate (TPA)-induced ERK activation and SRF/ELK interaction (Fig. 4H). These data suggest that the formation of the SRF/ELK complex and consequent transcriptional activation require SRF phosphorylation by NLK and ELK phosphorylation by ERK at the same time.

### Deficiency in NLK enhances the MKL/SRF response in human and mouse cells

**Fig. 5:**
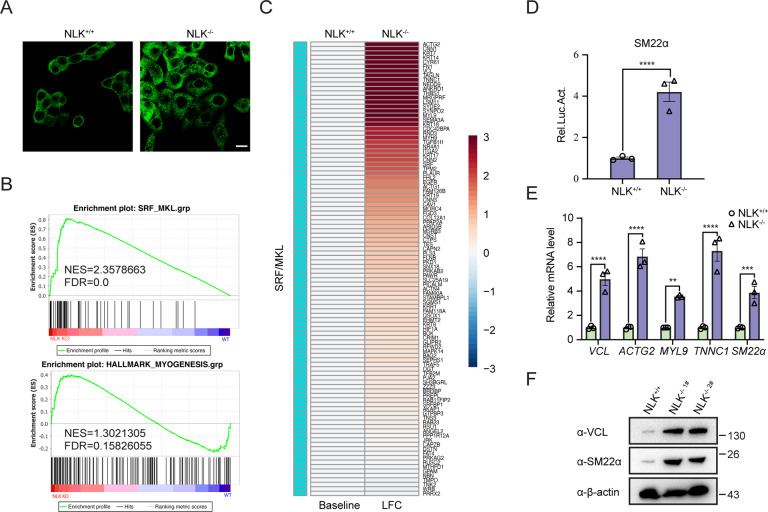
Loss of NLK promotes SRF/MKL signaling in HCT116 cells. **A** Immunofluorescence comparing cell size between wild-type and NLK-deficient HCT116 cells using an anti-p65 antibody. The cell cytosol (green) was stained using the anti-p65 antibody. Images were obtained by fluorescence microscopy. Scale bar, 10 μm. **B** GSEA showing SRF/MKL and myogenesis signaling enrichment between wild-type and NLK-deficient HCT116 cells. **C** Heatmap showing the differentially expressed genes involved in SRF/MKL signaling between wild-type and NLK-deficient HCT116 cells. **D** Luciferase assays showing the effects of NLK deficiency on SM22α in HEK293T cells. SM22α-reporter (100 ng) was transfected into HEK293T cells for 36 h, followed by analysis with a luciferase kit assay (*n* = 3). **E** Real-time PCR showing the gene transcription levels of *VCL, ACTG2, MYL9, TNNC1,* and *SM22α* in NLK-deficient HCT116 cells compared with wild-type cells. The real-time PCR values were normalized to the *GAPDH* values (*n* = 3). **F** Immunoblotting showing the protein levels of VCL, NLK, and SM22α in NLK-deficient HCT116 cells. Proteins were isolated from wild-type and NLK-deficient HCT116 cells, followed by immunoblotting experiments using the indicated antibodies. β-actin was used as a loading control. Data are representative of three independent experiments and presented as the mean ± SEM. Statistical significance was analyzed by Student’s *t*-test or ANOVA (**p < 0.01, ****p < 0.0001). LFC log fold change, NES normalized enrichment score, FDR false discovery rate. Source data (**A–F**) are provided as a source data file.

Given that ELK/SRF promotes cell growth, we observed that NLK deficiency significantly inhibited cell cycle progression and cell growth in colorectal cancer [20]. Another more interesting phenotype was that NLK-deficient-HCT116 cells displayed an enlarged cell morphology compared to HCT116 cells (Fig. 5A); however, we did not notice a cell size change by flow-cytometry analysis (Extended Data Fig. 1A). This finding indicated that NLK deficiency resulted in cell spreading. Moreover, we noticed enhanced reattachment from suspension in NLK-deficient-HCT116 cells compared to HCT116 cells (Extended Data Fig. 1B). These phenotypes captured our attention because SRF/MKL-regulated cytoskeletal genes are well documented to be involved in cell morphogenetic and adhesive processes [21, 22]. Intriguingly, previous studies have shown that SRF controls the proliferation and contractility balance through its cofactor complexes ELK/SRF and MKL/SRF [9]. Thus, we carried out GSEA and heatmap assays to determine the changes in MKL/SRF signaling. GSEA showed significant positive enrichment in the MKL/SRF target gene set in NLK-deficient HCT116 cells compared to the wild-type control cells, and the log2-fold change in expression data are shown in the heatmap (Fig. 5B, C). The transcriptional activity of SM22α, an MKL/SRF target, was dramatically increased in NLK-deficient-HCT116 cells (Fig. 5D). The mRNA levels of the MKL/SRF target genes *VCL, TNNC1, SM22α, ACTG2,* and *MYL9* were detected and consistent with the RNA-seq results (Fig. 5E). Consistently, the protein levels of VCL and SM22*α* were also upregulated in NLK-deficient cells (Fig. 5F). Taken together, these data suggest that NLK regulates MKL/SRF signaling in human cells.

### NLK inhibits the MKL/SRF response and differentiation in mouse myoblasts

**Fig. 6:**
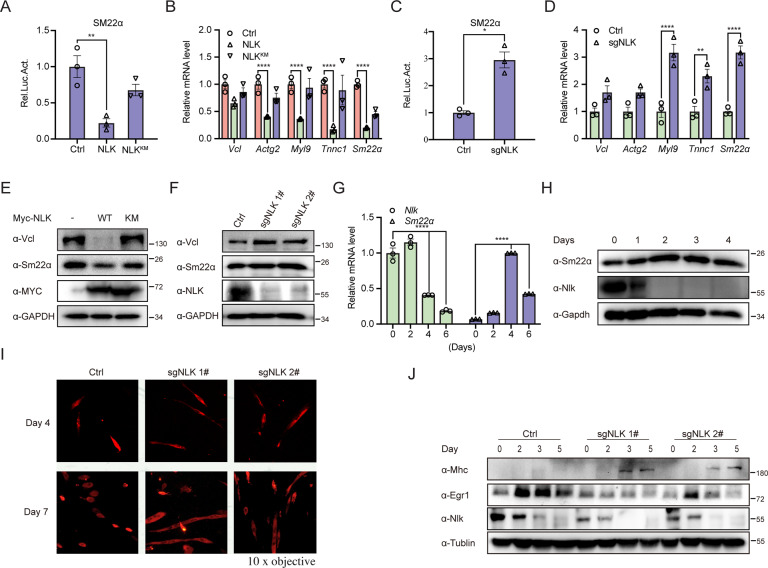
NLK regulates muscle differentiation by promoting SRF/MKL signaling in C2C12 cells. **A** Luciferase assays showing the effects of NLK or NLK^KM^ on SM22α in C2C12 cells. SM22α-reporter (200 ng) was cotransfected with an NLK or NLK^KM^ plasmid (200 ng) into C2C12 cells for 36 h, followed by analysis with a luciferase kit assay (*n* = 3). **B** Real-time PCR showing the effects of NLK or NLK^KM^ on *Vcl, Actg2, Myl9, Tnnc1,* and *Sm*22α gene transcription. The NLK or NLK^KM^ plasmid (200 ng) was transfected into C2C12 cells for 36 h, followed by real-time PCR experiments. The real-time PCR values were normalized to the *Gapdh* values (*n* = 3). **C** Luciferase assays with SM22α-reporter in wild-type and NLK-knockdown C2C12 cells. SRF/ELK reporter (200 ng) was transfected into NLK-knockdown C2C12 cells for 36 h, followed by analysis with a luciferase kit assay (*n* = 3). **D** Real-time PCR showing the gene transcription levels of *Vcl, Actg2, Myl9, Tnnc1,* and *Sm*22α in wild-type and NLK-knockdown C2C12 cells. The real-time PCR values were normalized to the *Gapdh* values (*n* = 3). **E** Immunoblotting showing the effects of NLK or NLK^KM^ on the protein levels of Vcl and Sm22α using the indicated antibodies in C2C12 cells. GAPDH was used as a loading control. **F** Immunoblotting showing the protein levels of Vcl and Sm22α in NLK-knockdown C2C12 cells compared with wild-type cells using anti-Vcl and anti-Sm22α antibodies in wild-type and NLK-knockdown C2C12 cells. GAPDH was used as a loading control. **G** Real-time PCR showing the gene transcription levels of *Nlk and Sm*22α in horse serum-induced C2C12 cells at the indicated times. The real-time PCR values were normalized to the *Gapdh* values (*n* = 3). **H** Immunoblotting showing the protein levels of Nlk and Sm22α in horse serum-induced C2C12 cells at the indicated times. Proteins were isolated from horse serum-induced C2C12 cells at the indicated times, followed by immunoblotting experiments using anti-Nlk and anti-Sm22α antibodies. Gapdh was used as a loading control. **I** Immunostaining for MHC in control and NLK-knockdown C2C12 cells on the 4th and 7th days after horse serum-mediated differentiation induction. **J** Immunoblotting showing the protein levels of Mhc, Srf, Elk1, Egr1, and Nlk in horse serum-induced C2C12 cells compared with NLK-knockdown C2C12 cells at the indicated times. Proteins were isolated from horse serum-induced C2C12 cells and NLK-knockdown C2C12 cells at the indicated times, followed by immunoblotting experiments using the indicated antibodies. Tubulin was used as a loading control. Data are representative of three independent experiments and presented as the mean ± SEM. Statistical significance was analyzed by ANOVA (*p < 0.05, **p < 0.01, ****p < 0.0001). Ctrl control. Source data (**A–J**) are provided as a source data file.

Since the SRF/MKL complex is a key regulator of muscle-specific genes, we next set out to determine the effects of NLK on the SRF/MKL pathway in mouse C2C12 myoblasts. Overexpression of NLK inhibited the transcriptional activity of SM22*α* and the mRNA transcription of MKL/SRF downstream genes (Fig. 6A, B). In contrast, NLK deficiency increased the transcriptional activity of Sm22*α* and the mRNA transcription of the MKL/SRF downstream genes *Vcl, Tnnc1, Sm22α, Actg2,* and *Myl9* (Fig. 6C, D). The protein levels of Vcl and Sm22*α* in Nlk-overexpressing and Nlk-deficient C2C12 cells were consistent, which clearly showed that NLK inhibited the MKL/SRF pathway in mouse C2C12 cells (Fig. 6E, F).

Given that NLK potently inhibits SRF/MKL signaling, we hypothesized that NLK may serve as a negative regulator of myoblast differentiation. To this end, horse serum and high-confluence cells were employed to induce the differentiation of C2C12 cells [23, 24]. We detected mRNA and protein levels during the differentiation course of C2C12 cells. Intriguingly, while the mRNA and protein levels of the differentiated C2C12 marker Sm22*α* were increased, both the mRNA and protein levels of Nlk were decreased during myoblast differentiation progression, indicating a negative correlation between NLK expression and myoblast differentiation (Fig. 6G, H). Furthermore, Nlk knockdown resulted in more MHC-positive myotubes with positive muscle cell-specific protein marker MHC immunostaining on days 4 and 7 of induction (Fig. 6I). For quantitative analysis, we assessed the protein levels of components of the MHC with immunoblotting assays. A relative expression delay for MHC and reduced expression of SRF/ELK pathway component Egr1 was observed during muscle differentiation in Nlk-knockdown cells compared to parental cells (Fig. 6J). Taken together, these data suggest that NLK is a negative regulator of SRF/MKL signaling and muscle cell differentiation as well as cell morphology in mouse C2C12 cells.

### Phosphorylation of SRF at residues 101/103 by NLK modulates the balance between SRF/ELK and SRF/MKL

**Fig. 7:**
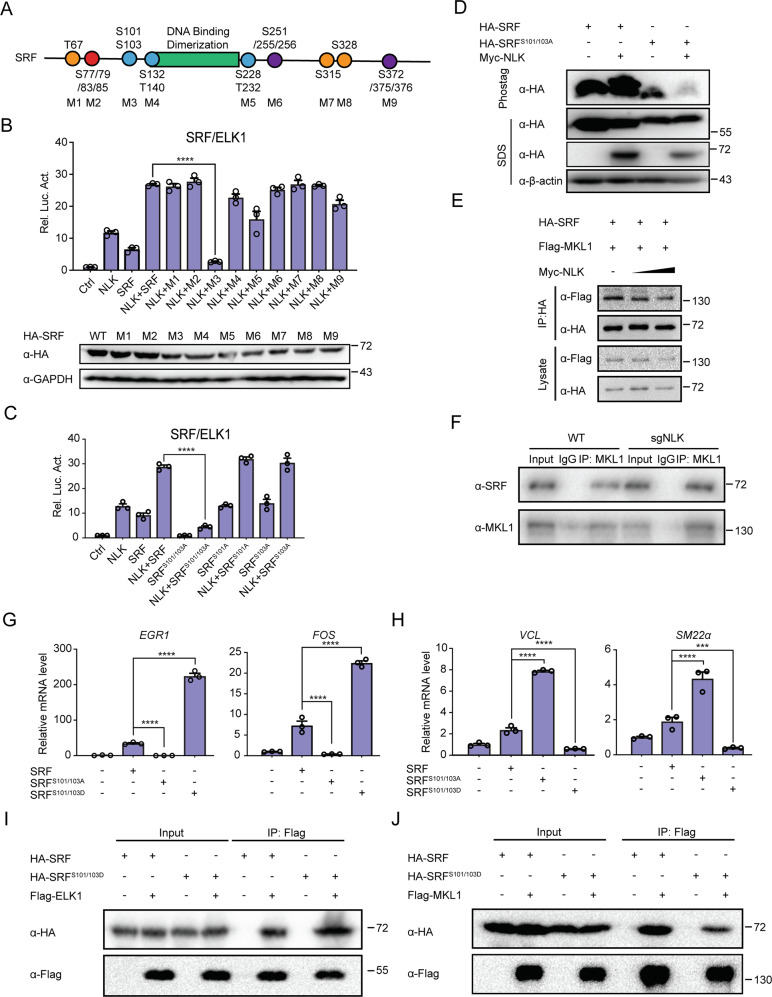
Phosphorylation of SRF at serines 101/103 inhibits SRF/MKL signaling. **A** Schematic diagram showing SRF serine and threonine sites. Orange: one site; blue: two sites; purple: three sites; and red: four sites. M1 to M9: serine or/and threonine to alanine mutants corresponding to indicated amino acid residues. **B** Luciferase assays analyzing the synergistic effects of M1 to M9 with NLK on SRF/ELK1 signaling in HEK293T cells. SRF/ELK1-reporter (200 ng) was cotransfected with an NLK or/and SRF wild-type and mutant plasmids (200 ng) into HEK293T cells for 36 h, followed by analysis with a luciferase kit assay (*n* = 3). Bottom line, Immunoblotting showing the protein levels of wild-type HA-SRF and mutant HA-SRF. Proteins were isolated from cells overexpressing SRF wild-type and mutant plasmids, followed by immunoblotting experiments using an anti-HA antibody. GAPDH was used as a loading control. **C** Luciferase assays analyzing the synergistic effects of SRF mutants (S101A, S103A, and S101A/S103A) and NLK on SRF/ELK1 signaling in HEK293T cells. SRF/ELK1-reporter (200 ng) was cotransfected with the NLK or/and SRF wild-type and mutant plasmids (200 ng) into HEK293T cells for 36 h, followed by analysis with a luciferase kit assay (*n* = 3). **D** Phos-tag^TM^ SDS-PAGE showing the phosphorylation statuses of SRF and the SRF^S101/103D^ mutant in the presence or absence of NLK in HEK293T cells. HA-SRF and the SRF^S101/103A^ mutant were cotransfected with Myc-NLK-overexpression plasmids into HEK293T cells, which were lysed after transfection for 36 h. Proteins were separated by SDS-PAGE containing Phos-tag^TM^ and general SDS-PAGE with anti-HA and anti-Myc antibodies. **E** Coimmunoprecipitation analysis showing the effects of NLK on the interaction between MKL1 and SRF. HEK293T cells were cotransfected with HA-SRF, Flag-MKL1, and Myc-NLK. Coimmunoprecipitation and immunoblotting were performed with the indicated antibodies. **F** Coimmunoprecipitation of endogenous proteins in wild-type and NLK-knockdown C2C12 cells performed using an anti-MKL1 antibody. Immunoblotting was performed with anti-MKL1 and anti-SRF antibodies. **G**, **H** Real-time PCR showing the effects of wild-type SRF, the SRF^S101/103D^ mutant or the SRF^S101/103A^ mutant on the gene transcription levels of *EGR1 and FOS* (**G**) or *SM22α* and *VCL* (**H**) in HEK293T cells. Total mRNA was isolated from SRF-, SRF^S101/103D^-, or SRF^S101/103A^-overexpressing HEK293T cells, and then reverse transcription and real-time PCR experiments were performed. The real-time PCR values were normalized to the *GAPDH* values (*n* = 3). **I**, **J** Coimmunoprecipitation analysis of the interaction between MKL1 (**I**) or ELK1 (**J**) and SRF or the SRF^S101/103D^ mutant. HEK293T cells were cotransfected with HA-SRF, HA-SRF^S101/103D^, Flag-MKL1, or Flag-ELK1. Coimmunoprecipitation and immunoblotting were performed with the indicated antibodies. Data are representative of three independent experiments and presented as the mean ± SEM. Statistical significance was analyzed by ANOVA (***p < 0.001, ****p < 0.0001). Ctrl control. Source data (**A–J**) are provided as a source data file.

To identify the phosphorylated site by NLK, we mutated some of serine/threonine in SRF to alanine according to the region distance span (Fig. 7A). Through luciferase-based screening, the serine 101 and 103 residues of SRF were found to play crucial roles in coordinating NLK-mediated activation of SRF/ELK transcription (Fig. 7B, C). Furthermore, a Phos-tag assay confirmed that NLK phosphorylated SRF at the serine 101 and 103 residues (Fig. 7D).

Previous studies have shown a balance between SRF/ELK and SRF/MKL controlled by SRF; however, how SRF controls this balance remains elusive. Considering that NLK phosphorylates SRF, we hypothesized that NLK modulates the balance between SRF/ELK and SRF/MKL and subsequently regulates muscle cell differentiation by phosphorylating SRF at the serine 101 and 103 residues. We showed that NLK increased the association between SRF and ELK. On the other hand, NLK deficiency decreased the binding between SRF and ELK1 in C2C12 cells. Therefore, whether NLK can reduce the association between SRF and MKL needs to be addressed. Coimmunoprecipitation showed that NLK overexpression reduced the association between SRF and MKL (Fig. 7E). Next, we sought to explore the function of constitutively phosphorylated SRF using a mutant in which the serine residues at sites 101/103 were changed to aspartic acid (SRF^S101/103D^). The results showed that SRF^S101/103D^ increased the binding to ELK and associated transcriptional activity (Fig. 7G, I) and decreased the interaction to MKL and associated transcriptional activity (Fig. 7H, J). Overall, these data suggest that NLK phosphorylates SRF at residues 101/103, thereby modulating the balance between ELK and MKL.

### NLK deficiency enhances muscle hypertrophy

**Fig. 8:**
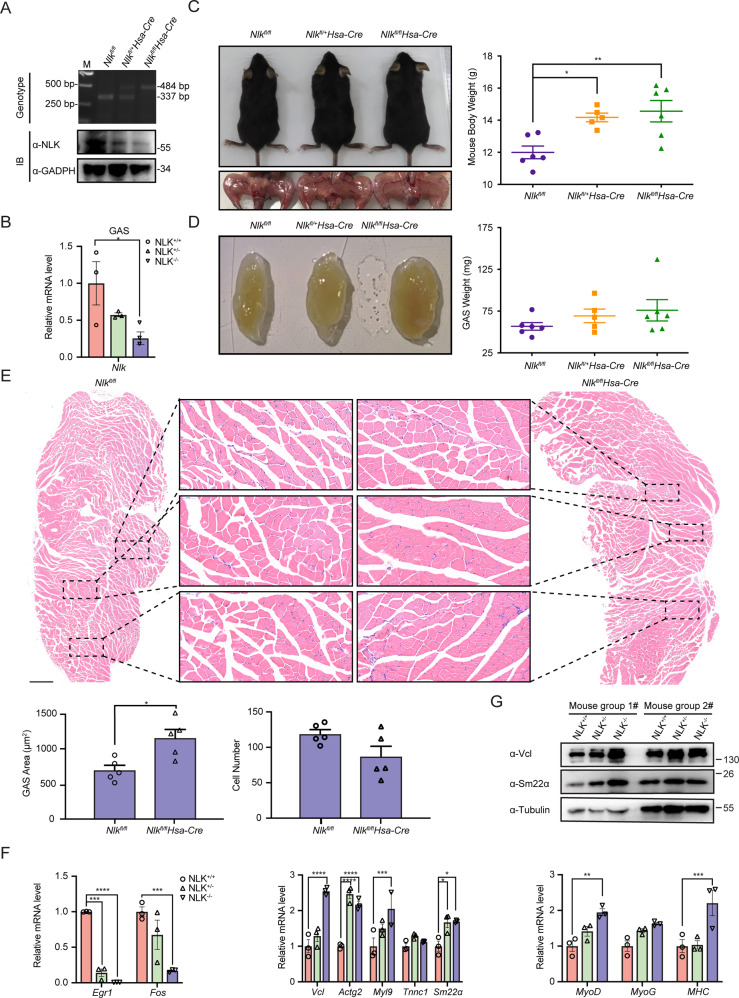
Conditional NLK knockout leads to mouse skeletal muscle hypertrophy. **A**, **B** Identification of mice with conditional skeletal muscle deletion (Nlk^fl/fl^HSA-Cre) at the genome, protein (**A**) and mRNA levels (**B**) via genomic PCR, immunoblotting and real-time PCR, respectively (*n* = 3). **C**, **D**, Mouse, leg (**C**) and skeletal muscle images (**D**) as well as corresponding graphical representation showing increased whole body and skeletal muscle weights for Nlk-deficient mice compared with wild-type mice (*n* = 6). **E** Hematoxylin and eosin staining and the corresponding graphical representation showing increased skeletal muscle cell numbers in Nlk-deficient mice compared with wild-type mice. Scale bar, 500 μm. **F** Real-time PCR showing the gene transcription levels of *Egr1, Fos, Vcl, Actg2, Myl9, Tnnc1, Sm22α, MyoG, MyoD,* and *MHC* in wild-type and Nlk-deficient mouse skeletal muscle. The real-time PCR values were normalized to the *Gapdh* values (*n* = 3). **G** Immunoblotting showing the protein levels of Vcl and Sm22α in Nlk-deficient mouse skeletal muscle compared with wild-type mouse skeletal muscle using the indicated antibodies. Gapdh was used as a loading control. Data are representative of three independent experiments and presented as the mean ± SEM. Statistical significance was analyzed by ANOVA (*p < 0.05, **p < 0.01, ***p < 0.001, ****p < 0.0001). IB immunoblotting, GAS gastrocnemius. Source data (**A–G**) are provided as a source data file.

C57BL/6 mice lacking Nlk die during the third trimester of pregnancy [25]. To elucidate the roles of NLK in skeletal muscle development and maturation in vivo, we used previously generated *Nlk*^*fl/fl*^ mice [26] and bred these mice with transgenic mice expressing the Cre recombinase gene driven by the muscle-specific human α-skeletal actin (HSA) promoter [27] to generate skeletal muscle-specific *Nlk* conditional knockout mice (*Nlk*^*fl/fl*^*/HSA-Cre*). Genotypes were evaluated by genomic PCR, which showed different genotypes for the wild-type and *Nlk* skeletal muscle-specific depletion mice (Fig. 8A). We further verified the mRNA and protein levels of Nlk in the gastrocnemius (GAS). Real-time PCR and western blotting confirmed the reduced expression of *Nlk* in *Nlk*^*fl/fl*^*/HSA-Cre* mice compared to wild-type mice (Fig. 8A, B).

Because HSA is active during the later period in the pathway of skeletal muscle development [27], we wondered whether ablation of Nlk in skeletal muscle at a later time might produce a distinctive phenotype. We measured the bodyweight of mice at 0 or 4 weeks of age. Strikingly, the bodyweight of the *Nlk-*ablation mice showed a significant increase compared to that of the wild-type mice at 4 weeks (Fig. 8C); however, there was no difference in bodyweight at birth. We also examined the weight of the GAS. As expected, the weight of this skeletal muscle increased in Nlk skeletal muscle-specific depletion mice compared to wild-type mice (Fig. 8D). This indicated that Nlk is involved in postnatal muscle mass increase because the deletion of Nlk was specific to the skeletal muscle. To investigate the role of Nlk in skeletal muscle hypertrophy, we further observed the area and number of myofibers by hematoxylin and eosin (H&E) staining of the GAS. A clearly increased myofiber cross-sectional area was observed; however, the myofiber number was slightly decreased (Fig. 8E), which indicated skeletal muscle hypertrophy in *Nlk*^*fl/fl*^*/HSA-Cre* mice. Furthermore, the downregulation of SRF/ELK downstream gene expression and upregulation of SRF/MKL downstream gene expression were examined (Fig. 8F, G). Altogether, these data suggest that Nlk is a crucial regulator required for skeletal muscle hypertrophy that functions by tuning the SRF/ELK and SRF/MKL pathways.

## Discussion

RAS/ERK-ELK/SRF mediate a number of genes involved in cell proliferation and growth. In general, RAS is activated in response to the binding of extracellular signals, such as growth factors, with binding followed by a linear cascade through RAF, MEK, and ERK [28]. Phosphorylated ERK moves into the nucleus and phosphorylates ELK, which results in enhanced binding between ELK and SRF and transactivation of a batch of specific target genes. Phosphorylation of ELK at serine 383 is required for activation by ERK; however, it is unclear whether the phosphorylation event of SRF is involved in SRF/ELK signaling. Thus, we screened ~200 kinases to identify potential candidate proteins that play important roles in ELK/SRF activation. NLK, an evolutionarily conserved serine/threonine-protein kinase that displays strong activation effects on the ELK/SRF pathway due to its high similarity to ERK, caught our attention.

First, we identified a transactivation effect by overexpressing-NLK in 293 T cells. We further confirmed this transcriptional activation using previously generated NLK-deficient-HCT116 cells. Moreover, NLK overexpression and NLK ablation in mouse C2C12 cells verified the effects of NLK on the ELK/SRF pathway. Considering the similarity between NLK and ERK, the binding of NLK and ELK was explored. However, no association was detected between NLK and ELK. Instead, the same downstream transcription factor SRF was found to exhibit strong binding with NLK. Next, the phosphorylation of SRF by NLK was proven using Phos-tag^TM^ SDS-PAGE. Based on the facts that NLK phosphorylates SRF and enhances ELK/SRF binding and that NLK is required for ELK/SRF signaling, we propose that the NLK-SRF and ERK-ELK axes are parallel pathways that orchestrate ELK/SRF activation.

**Fig. 9:**
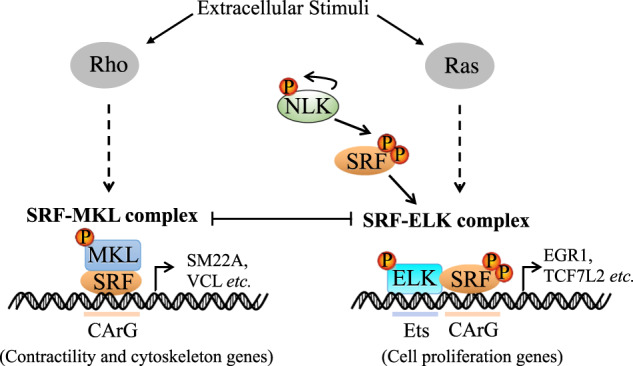
Model of NLK/SRF/ELK Pathway. Work model of NLK regulating the balance between SRF/ELK and SRF/MKL through phosphorylating SRF.

Observation of NLK-deficient-HCT116 cells revealed that the morphology of these cells was clearly changed from that of parental cells. Coactivator TCFs and MKLs compete for binding to SRF to regulate cell proliferation and contractility, respectively [9]. This suggests that in addition to regulating the SRF/ELK pathway, SRF phosphorylation by NLK is also involved in SRF/MKL signaling. We proved this in NLK-deficient-HCT116 cells and mouse C2C12 cells. In vitro differentiation models using C2C12 myoblasts displayed reduced Nlk expression, further confirming that Nlk is involved in muscle differentiation. In vivo, muscle-specific Nlk depletion resulted in muscle hypertrophy and upregulation of the SRF/MKL pathway as well as dramatic downregulation of the SRF/ELK pathway. Overall, we identified a new NLK-SRF axis that is required for ERK-ELK/SRF signal transduction. Phosphorylated SRF not only enhances the association of SRF and ELK and transactivation but also reduces binding between SRF and MKL and associated signal transduction as well as subsequent muscle development (Fig. 9). Thus, NLK plays an important role in controlling the balance between cell proliferation and contractility by phosphorylating SRF, which is crucial for maintaining muscle growth and differentiation homeostasis.

Phosphorylation plays a role and often turns signaling on/off. The N-terminal phosphorylation sites of SRF were studied by Ralf Janknecht et al. who reported that replacing serine 103 with alanine in SRF could decrease DNA-binding activity. Our results showed that serine 101/103 phosphorylation could increase the activity of SRF/ELK, but serine 103 phosphorylation alone could not. The potential explanation is that the cell-free system used in that study results in the identified difference. Replacement of serine 101/103 in SRF with alanine almost completely blocked SRF/ELK transcriptional activity, indicating that serine 101/103 phosphorylation is quite important and required for SRF activity. Our experiments suggested that phosphorylation could switch the signaling pathway, which enhanced the understanding of the role of SRF phosphorylation sites.

NLK deficiency dramatically increased mouse muscle weight and hypertrophy, and this phenotype allowed us to define NLK as an inhibitory regulator of muscle development. Ablation of NLK gave rise to decreased SRF phosphorylation and enhanced SRF/MKL pathway activity. Deletion of Srf in skeletal muscle tissues results in a reduced whole bodyweight phenotype and death from an inability to breathe [1], which is consistent with the NLK-deficient phenotype. These similar results suggest the function of NLK in muscle tissue is associated with and dependent on Srf. It is difficult to detect the expression of Nlk in the muscle tissues of mice, suggesting that persistent aberrant expression of Nlk influences muscle quality and development. Therefore, Nlk is a critical factor for mouse muscle fate determination. Currently, we do not know whether exercise can further enhance muscle quality in Nlk-deficient mouse muscle tissues. However, it is a way to target Nlk in the early stage of muscle development to enhance muscle strengthening, which would also be helpful for athletes if the side effects could be well controlled. It was beyond our expectation that Nlk knockout in mouse muscle tissues would result in an increase in the whole bodyweight. Because Nlk knockout was specific to muscle tissues, we believed the increase in bodyweight to be an indirect result. Our opinion is that Nlk-deficiency-induced muscle function alterations affected whole body energy metabolism and eventually lead to bodyweight gain. Therefore, the role of NLK in energy metabolism still needs to be clarified in the future.

The RAS/ERK pathway has been intensely studied for approximately three decades. In summary, we identified that the new axis NLK-SRF is parallel with and required for Ras/ERK-ELK/SRF signaling activation. NLK phosphorylates SRF at the serine 101/103 residues, which are involved in skeletal muscle development and body energy metabolism. These findings further the understanding of the central role of SRF in regulating cell proliferation and contractility. The serine 101/103 residues of SRF could be a potential target for treatment in muscle development-associated diseases.

## Methods

### Cell lines and mice

All the cell lines used in the study were originally derived from the Chinese Academy of Sciences Cell Bank (Shanghai, China). And no mycoplasma contamination was detected. NLK^−/−^ HCT116 cells were previously generated via AAV-mediated gene knockout procedures [17, 20]. To obtain Nlk-knockdown C2C12 cells, sgRNAs specific for Nlk were constructed and ligated into a lentiCRISPR v2 vector, which was cotransfected with the pGag-pol and pVSV-G plasmids into HEK293T cells. Two days after transfection, the viruses were harvested and used to infect C2C12 cells in the presence of polybrene (8 µg/mL). The infected cells were selected via puromycin (1 µg/mL) treatment for 7 days. Given the effect size and standard deviation, the method for determining the sample size in animal studies was chosen in accordance with the animal research committee’s suggestion. The mice were randomly divided into each group. C57BL/6 NLK conditional knockout mice were previously generated [26]. HSA-Cre mice were obtained from The Jackson Laboratory (B6.Cg-Tg(ACTA1-cre)79Jme/J, Stock No: 006149). Four-week-old male and female C57BL/6 mice were used for the experiments. All mice were housed under a 12:12 h light/dark cycle at a controlled temperature. All animal studies were conducted in accordance with the Guidelines of the China Animal Welfare Legislation and approved by the Committee on Ethics in the Care and Use of Laboratory Animals of Wuhan University. All efforts were made to minimize suffering.

### Reporter assays

HEK293T cells were transfected with plasmids encoding the indicated luciferase reporters, pRL-TK *Renilla* luciferase, and different expression or control vectors using TurboFect reagent (Thermo Scientific, Waltham, MA, USA). Twenty-four to thirty-six hours later, a dual-reporter specific luciferase assay kit (Promega, Madison, WI, USA) was used to evaluate luciferase activity.

### H&E staining and immunofluorescence

Obtained GAS muscle tissue was fixed in 4% paraformaldehyde and embedded in paraffin. Paraffin-embedded tissues were cut into 4 μm sections and heated at 60 °C for 45 min before a standard deparaffinization process was performed. H&E were then used for staining. For immunofluorescence, cells were fixed in 4% paraformaldehyde for 10 min and permeabilized with 0.25% Triton X-100 in PBS for 15 min at room temperature, followed by washing in PBS. Next, the cells were blocked with 3% bovine serum albumin (BSA) in PBS for 30 min and incubated with a primary antibody (diluted with 3% BSA in PBS) overnight at 4 °C. Then, the samples were incubated with a secondary antibody (diluted with 3% BSA in PBS) for 1 h at room temperature before staining with DAPI in the dark at 37 °C for 10 min. The coverslips were mounted on glass slides with an antifade solution. The slides were imaged using a confocal microscope.

### C2C12 cell differentiation

C2C12 murine skeletal muscle myoblasts were seeded in six-well plates in DMEM supplemented with 20% FBS (Gibco, Shanghai, China) and then further cultured in fusion medium (DMEM supplemented with 2% horse serum) when the cells grew to ~90% confluent. Approximately 4–7 days, multicore microtubes could be observed in the plate by microscopy.

### RNA isolation and quantitative PCR

Cells were lysed with TRIzol (TAKARA, Otsu, Japan), and RNA was isolated according to standard protocols. Typically, ~1 μg of total RNA was used to make complementary DNA through reverse transcription according to the manufacturer’s instructions (Thermo Scientific, Waltham, MA, USA). The quantities of specific mRNA transcripts were determined by quantitative PCR. All real-time PCR values were normalized to *GAPDH* mRNA expression values. The oligonucleotides used in the study are presented in Supplementary Table 1.

### GSEA analyses

GSEA was performed using GSEA 4.0.3 (https://www.gsea-msigdb.org/gsea/index.jsp) according to the user manual. RNA-seq results were preranked based on the log2-fold change to generate an RNK file. The preranked list was used to calculate the enrichment score (ES) via a weighted Kolmogorov-Smirnov–like statistic of GSEA. The statistical significance (nominal *p*-value) of the ES was determined by the distribution of ESs from a 1000-permutations analysis of gene sets. Normalized enrichment scores (NES) and false discovery rate (FDR) q-values were calculated using default GSEA parameters.

### Coimmunoprecipitation and immunoblot analyses

Whole-cell extracts were generated by incubating cells in NP-40 lysis buffer (30 mM Tris-HCl pH 7.4, 150 mM NaCl, and 1% NP-40) supplemented with a protease and phosphatase inhibitor cocktail (Roche, Basel, Switzerland) on ice for 30 min, followed by a brief sonication to ensure nucleus rupture. After centrifugation for 15 min at 12,000 × *g*, the supernatants were incubated with immunoprecipitation antibodies and Protein A/G beads (Roche) overnight at 4 °C. The beads were washed 3–5 times with wash buffer (30 mM Tris-HCl pH 7.4, 150 mM NaCl, and 0.1% NP-40) before the immunoprecipitates were boiled in 1× SDS loading buffer (50 mM Tris-HCl pH 6.8, 10% glycerol, 1% SDS, and 1% β-glycerophosphate) at 95 °C for 10 min and separated on 10–15% SDS-PAGE gels. The proteins were transferred to PVDF membranes (Bio-Rad, Hercules, CA, USA) and then incubated with primary antibodies. The Clarity™ Western ECL Substrate System (Bio-Rad) was used for protein detection. The details of the primary antibodies are provided in Supplementary Table 2.

### Phos-tag^TM^ SDS-PAGE assays

Cells were lysed in NP-40 lysis buffer supplemented with a protease and phosphatase inhibitor cocktail (Roche, Basel, Switzerland). Next, SDS loading buffer was added to the supernatants, which were separated on SDS-PAGE gels containing a 50 µmol/L Phos-tag solution (Wako, Japan) and 100 µmol/L MnCl_2_. Electrophoresis was performed at a constant current of 30 mA/gel. Before the transfer to PVDF membranes performed on ice for 6 h, the gels were washed three times in wash buffer (containing 10 mM EDTA) for 10 min and once in transfer buffer for 10 min to remove metal ions.

### Statistical analysis

Data are expressed as the mean ± SEM. Statistical significance was evaluated using an unpaired two-tailed Student’s *t*-test or ANOVA (more than two groups) by Prism software (Version 8.0). Differences were considered significant at a *P*-value < 0.05.

## Supplementary information

Supplementary Table 1

Supplementary Table 2

Extended Figure 1 legend

Extended Data Figure 1

## Data Availability

The authors declare that all data supporting the findings of this investigation are available within the article, its Supplementary Information, and from the corresponding authors upon reasonable request.
