# Supplementary material for: NLK is required for Ras/ERK/SRF/ELK signaling to tune skeletal muscle development by phosphorylating SRF and antagonizing the SRF/MKL pathway
Source: Cell Death Discov. 2022 Jan 10;8:4. doi: 10.1038/s41420-021-00774-9 (PMC8748963; doi:10.1038/s41420-021-00774-9)
Supplement: Supplementary file 1 — Supplementary Table 1 [file 41420_2021_774_MOESM1_ESM.pdf]

Genes	Primers	Primer sequences
GAPDH	Forward	5'-GAGTCAACGGATTTGGTCGT-3'
	Reverse	5'-GACAAGCTTCCCGTTCTCAG-3'
EGR1	Forward	5'-GGTCAGTGGCCTAGTGAGC-3'
	Reverse	5'-GTGCCGCTGAGTAAATGGGA-3'
FOS	Forward	5'-CCGGGGATAGCCTCTCTTACT-3'
	Reverse	5'-CCAGGTCCGTGCAGAAGTC-3'
TCF7L2	Forward	5'-AGAAACGAATCAAAACAGCTCCT-3'
	Reverse	5'-CGGGATTTGTCTCGGAAACT-3'
NLK	Forward	5'-CGCAAAAATGATGGCGGCTTA-3'
	Reverse	5'-CCCAGGGTTTAACATGGCTG-3'
VCL	Forward	5'-CTCGTCCGGGTTGGAAAAGAG-3'
	Reverse	5'-AGTAAGGGTCTGACTGAAGCAT-3'
ACTG2	Forward	5'-GCGTGTAGCACCTGAAGAG-3'
	Reverse	5'-GAATGGCGACGTACATGGCA-3'
MYL9	Forward	5'-TCTTCGCAATGTTTGACCAGT-3'
	Reverse	5'-GTTGAAAGCCTCCTTAAACTCCT-3'
TNNC1	Forward	5'-TGGTTCGGTGCATGAAGGAC-3'
	Reverse	5'-GTCGATGTAGCCATCAGCATT-3'
SM22α	Forward	5'-AGTGCAGTCCAAAATCGAGAAG-3'
	Reverse	5'-CTTGCTCAGAATCACGCCAT-3'
Nlk	Forward	5'-CACAAGAGCCAACGCAAAAAT-3'
	Reverse	5'-TGCCGAAGACGTATGCTGC-3'
Egr1	Forward	5'-TCGGCTCCTTTCCTCACTCA-3'
	Reverse	5'-CTCATAGGGTTGTTCGCTCGG-3'
Fos	Forward	5'-CGGGTTTCAACGCCGACTA-3'
	Reverse	5'-TGGCACTAGAGACGGACAGAT-3'
Tcf7l2	Forward	5'-TCATCACGTACAGCAATGAACA-3'
	Reverse	5'-CGACAGCGGGTAATATGGAGAG-3'
Vcl	Forward	5'-TCTCGCACCTGGTGATTATGC-3'
	Reverse	5'-TGAACAGTCTCTTTTCCAACCC-3'
Actg2	Forward	5'-CCGCCCTAGACATCAGGGT-3'
	Reverse	5'-TCTTCTGGTGCTACTCGAAGC-3'
Myl9	Forward	5'-AGAGGGCTACGTCCAATGTCT-3'
	Reverse	5'-CTCCAGATACTCGTCTGTGGG-3'
Tnnc1	Forward	5'-GCGGTAGAACAGTTGACAGAG-3'
	Reverse	5'-GACAAGAAACTCATCGAAGTCCA-3'
Sm22α	Forward	5'-GCTATGGCATTAACACCACGG-3'
	Reverse	5'-CCCAGGTTCATTAGTGTCCGC-3'
MyoD	Forward	5'-CTTTGCTTACGTCAGTCAAGGT-3'
	Reverse	5'-AGCGCCTGTGAGCTTGTAAA-3'
MyoG	Forward	5'GCTGCTTATCTGACAAGTCTGAA3'
	Reverse	5'GGCCTTTGGTTACGAACTCATT3'
MHC	Forward	5'-GAGACATCCCCCTATTTCTACCA-3'

MHC	Reverse	5'-GCTCAGTCCGCTCATAGCC-3'
mflox	Forward	5′-CTGTAACCACGAGGACAGCA-3′
	Reverse	5′-TTCTCCCTCCCTCTCTCACA-3′
HSA-Cre	Forward	5'-GCGGTCTGGCAGTAAAAACTATC-3'
	Reverse	5'-GTGAAACAGCATTGCTGTCACTT-3'
HSA-Cre	Forward	5'-CTAGGCCACAGAATGAAAGATCT-3'
	Reverse	5'-GTAGGTGGAAATTCTAGCATCATCC-3'
sgNlk1	Forward	5'-ATGATGGCGGCTTACAATGG-3’
	Reverse	5’-CCATTGTAAGCCGCCATCAT-3’
sgNlk2	Forward	5’-ATTGAGCCGGATAGACCTAT-3’
	Reverse	5’-ATAGGTCTATCCGGCTCAAT-3’

Application
RNA expression
RNA expression
RNA expression
RNA expression
RNA expression
RNA expression
RNA expression
RNA expression
RNA expression
RNA expression
RNA expression
RNA expression
RNA expression
RNA expression
RNA expression
RNA expression
RNA expression
RNA expression
RNA expression
RNA expression
RNA expression
RNA expression
RNA expression
RNA expression
RNA expression
RNA expression
RNA expression
RNA expression
RNA expression
RNA expression
RNA expression
RNA expression
RNA expression
RNA expression
RNA expression
RNA expression
RNA expression
RNA expression
RNA expression
RNA expression
RNA expression
RNA expression
RNA expression

RNA expression
Genome typing
Genome typing
Genome typing
Genome typing
Genome typing
Genome typing
Gene deletion
Gene deletion
Gene deletion
Gene deletion
