# Supplementary material for: NLK is required for Ras/ERK/SRF/ELK signaling to tune skeletal muscle development by phosphorylating SRF and antagonizing the SRF/MKL pathway
Source: Cell Death Discov. 2022 Jan 10;8:4. doi: 10.1038/s41420-021-00774-9 (PMC8748963; doi:10.1038/s41420-021-00774-9)
Supplement: Supplementary file 2 — Supplementary Table 2 [file 41420_2021_774_MOESM2_ESM.pdf]

S.No.	Antibodies	Source	Antibody dilution	Identifier
1	Rabbit anti-β-Tubulin, polyclonal	Cell Signaling Technology	1:1000（WB）	Cat# 2146
2	Mouse anti-β-actin, monoclonal	MBL	1:1000（WB）	Cat# M177-3
3	Mouse anti-GAPDH, monoclonal	MBL	1:1000（WB）	Cat# M171-3
4	Mouse anti-NLK, polyclonal	Bethyl	1:500 (WB)	Cat# A400-046A
5	Mouse anti-MHC, monoclonal	DSHB	1:500 (WB)	Cat# MF 20
6	Rabbit anti-ERG1, monoclonal	Cell Signaling Technology	1:1000（WB）	Cat# 4154
7	Rabbit anti-VCL,polyclone	Cell Signaling Technology	1:1000（WB）	Cat# 4650
8	Rabbit anti-SM22α, polyclonal	Proteintech Group	1:1000（WB）	Cat# 10493-1-AP
9	Rabbit anti-MKL1,polyclone	ABclonal	1:1000（WB）1:100 (IP)	Cat# A8504
10	Rabbit anti-ELK1,polyclone	ABclonal	1:1000（WB）1:200 (IP)	Cat# A0789
11	Mouse anti-SRF, monoclonal	Proteintech Group	1:1000（WB）1:200 (IP)	Cat# 66742-1-Ig
12	Mouse anti-Flag, monoclonal	MBL	1:10000(WB) 1:500 (IP)	Cat# M185-3
13	Mouse anti-Myc, monoclonal	MBL	1:10000(WB) 1:500 (IP)	Cat# M192-3
14	Mouse anti-HA, monoclonal	MBL	1:750 (IF) 1:10000 (WB) 1:500 (IP)	Cat# M180-3
15	anti-human p65	Cell Signaling Technology	1:1000（WB）1:100 (IP)	Cat# 6956
16	Rabbit IgG HRP Linked Antibody	Jackson ImmunoResearch	1:10000	Cat# 111-035-003
17	Mouse IgG HRP Linked Antibody	Jackson ImmunoResearch	1:10000	Cat# 115-035-003
