# Supplementary material for: NLK is required for Ras/ERK/SRF/ELK signaling to tune skeletal muscle development by phosphorylating SRF and antagonizing the SRF/MKL pathway
Source: Cell Death Discov. 2022 Jan 10;8:4. doi: 10.1038/s41420-021-00774-9 (PMC8748963; doi:10.1038/s41420-021-00774-9)
Supplement: Supplementary file 3 — Extended Figure 1 legend [file 41420_2021_774_MOESM3_ESM.docx]

**Extended Figure 1. Loss of NLK promotes SRF/MKL signaling in HCT116 cells. a**, Flow cytometry analysis comparing cell size between wild-type and NLK-deficient HCT116 cells. Wild-type and NLK-deficient HCT116 cells were trypsinized and analyzed by flow cytometry. **b**, Analysis of the capacity for cell reattachment among the indicated cells after trypsinization. The indicated cells were trypsinized; seeded into plates; incubated for 0, 3 and 6 hours; and imaged using phase-contrast microscopy.
