# Supplementary figures and images for: NLK is required for Ras/ERK/SRF/ELK signaling to tune skeletal muscle development by phosphorylating SRF and antagonizing the SRF/MKL pathway
Source: Cell Death Discov. 2022 Jan 10;8:4. doi: 10.1038/s41420-021-00774-9 (PMC8748963; doi:10.1038/s41420-021-00774-9)

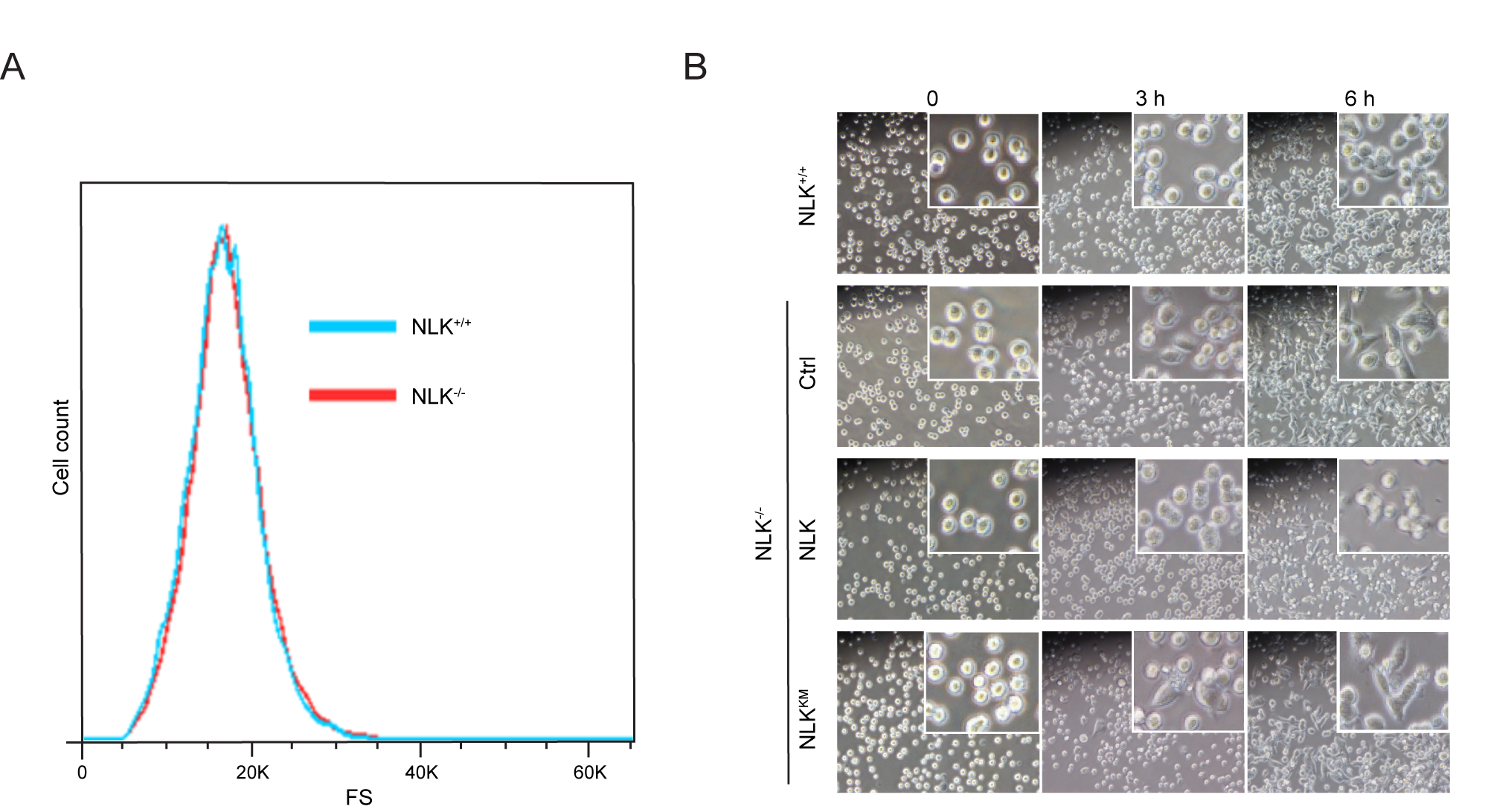

Supplement: Supplementary file 4 — Extended Data Figure 1 [file 41420_2021_774_MOESM4_ESM.tif]
